# Obesity and Inflammatory Factors in the Progression of Early-Onset Colorectal Cancer

**DOI:** 10.3390/cancers16071403

**Published:** 2024-04-03

**Authors:** Alexandra N. Jones, Katharina M. Scheurlen, Anne Macleod, Hillary L. Simon, Susan Galandiuk

**Affiliations:** 1 Price Institute of Surgical Research, University of Louisville, Louisville, KY 40202, USA; anjone18@louisville.edu (A.N.J.); anne.macleod@louisville.edu (A.M.); hillary.simon@louisville.edu (H.L.S.); 2 Division of Colon and Rectal Surgery, Hiram C. Polk Jr. MD Department of Surgery, University of Louisville, Louisville, KY 40202, USA

**Keywords:** obesity, early-onset colorectal cancer (EOCRC), cytokine-mediated inflammatory processes, tumor microenvironment, M1 macrophages, M2a macrophage, itaconate, pro-inflammatory cytokines, leptin, adiponectin, adipokines, microbiome

## Abstract

**Simple Summary:**

The incidence of colorectal cancer (CRC) in the United States (US) has decreased significantly over the last several decades, but primarily in those 65 years of age and older. However, there has been a dramatic global increase in the incidence of colorectal cancer in patients younger than 50 years old. This cohort is known as early-onset colorectal cancer (EOCRC) and is projected to rise in incidence. This epidemiologic change is poorly understood, but the sharp increase in the incidence of obesity and metabolic dysfunction have been linked to this phenomenon. The aim of our study is to examine the complex inflammatory mechanisms affecting EOCRC, including obesity, hormonal effects, inflammatory processes, and the tumor environment. With an improved understanding of these interactions, new methods to prevent and treat EOCRC could reduce its incidence and prolong survival in affected patients.

**Abstract:**

Metabolic dysfunction associated with obesity leads to a chronic pro-inflammatory state with systemic effects, including the alteration of macrophage metabolism. Tumor-associated macrophages have been linked to the formation of cancer through the production of metabolites such as itaconate. Itaconate downregulates peroxisome proliferator-activated receptor gamma as a tumor-suppressing factor and upregulates anti-inflammatory cytokines in M2-like macrophages. Similarly, leptin and adiponectin also influence macrophage cytokine expression and contribute to the progression of colorectal cancer via changes in gene expression within the PI3K/AKT pathway. This pathway influences cell proliferation, differentiation, and tumorigenesis. This work provides a review of obesity-related hormones and inflammatory mechanisms leading to the development and progression of early-onset colorectal cancer (EOCRC). A literature search was performed using the PubMed and Cochrane databases to identify studies related to obesity and EOCRC, with keywords including ‘EOCRC’, ‘obesity’, ‘obesity-related hormones’, ‘itaconate’, ‘adiponectin’, ‘leptin’, ‘M2a macrophage’, and ‘microbiome’. With this concept of pro-inflammatory markers contributing to EOCRC, increased use of chemo-preventative agents such as aspirin may have a protective effect. Elucidating this association between obesity-related, hormone/cytokine-driven inflammatory effects with EOCRC may help lead to new therapeutic targets in preventing and treating EOCRC.

## 1. Introduction

**Figure 1:**
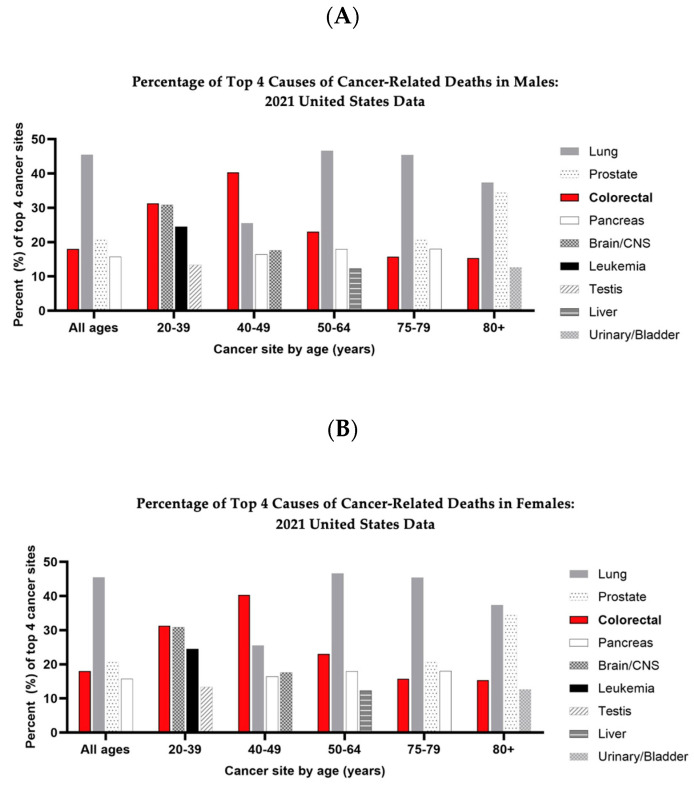
Top four causes of cancer-related deaths in the United States by age and sex, based on data obtained from Siegel et al. *Cancer Statistics*, 2024 [2] (**A**) The top four causes of cancer-related death in men in the United States by percentage. (**B**) The top four causes of cancer-related death in women in the United States by percentage.

Colorectal cancer (CRC) has the third-highest incidence of cancer diagnoses worldwide (8%) and contributes to 8–9% of cancer-related deaths, making it the third-most common cause of cancer deaths [1]. While the incidence of CRC in the United States (US) has decreased significantly over the last several decades, this phenomenon has been restricted primarily to the elderly. Conversely, there has been a dramatic global increase in the incidence of CRC in patients younger than 50 years old, known as early-onset colorectal cancer (EOCRC). This trend amongst the young is projected to continue to rise. Notably, the percentage of mortality among younger patients has also continued to increase (Figure 1) [2]. As a result of the rise in EOCRC, the United States Preventative Services Task Force (USPSTF) has issued a reduction in the recommended age, from 50 years of age to 45 years of age, for first-time CRC screening in average-risk patients [3,4]. This is not only a problem in the United States, but a global issue. In 2018, with an estimated 4.8 million new cases of cancer diagnoses and 3.4 million attributable deaths, gastrointestinal cancers (GI) (esophagus, stomach, colon–rectum, pancreas, liver) made up approximately 26% of incidence and 34% of deaths, with colorectal cancers making up approximately 1.8 million of those new cases. This made colorectal cancer the most diagnosed GI cancer worldwide [5]. Arnold et al. found an increasing incidence of colorectal cancer diagnoses was observed between 1960 and 2018 in previously low-incidence areas, i.e., Eastern Europe, Asia, and South America, secondary to changes in lifestyle and diet [5,6]. CRC rates in high-incidence areas such as Northern Europe, North America, and Canada continue to remain amongst the highest worldwide [5].

At the same time, obesity has now become an expanding public health concern, with over half of US and European populations meeting the criteria for being overweight or obese (BMI ≥ 25 kg/m^2^ for overweight and BMI ≥ 30 kg/m^2^ for obese) [7]. This has led to the suggestion of an association between metabolic dysfunction and carcinogenesis, as there has been a parallel rise in obesity and EOCRC since the 1950s. The development of obesity and poor dietary habits is believed to play strong roles in carcinogenesis [8,9]. Further supporting this association, the incidence of CRC is highest in western countries with high-fat diets, but it has also been shown to increase in conjunction with some other newly developing nations [3]. High-fat diets have been shown to upregulate the proliferator-activated receptor-delta (PPARδ) pathway, leading to alterations in intestinal progenitor stem cell function, as well increase their capacity to initiate tumor development in the gut [10]. The Nurses’ Health Study II, published in 2019, additionally showed an association between both body mass index and weight gain beginning at 18 years of age and an increased risk for EOCRC [11]. The links between these two trends continues to be evaluated, especially with new evidence demonstrating distinct molecular and genetic features of EOCRC compared to older-age onset [3,11]. A key hypothesis in where this link originates includes the idea that obesity elicits a chronic sub-inflammatory state, leading to macrophage polarization and a reduction of immunosuppressive cells such as natural killer and T cells [12,13]. 

The exact underlying mechanisms for this epidemiologic change, however, are poorly understood, which has resulted in an in-depth study of factors leading to the development and progression of EOCRC. One suggestion is the influence of obesity-related hormones, such as leptin and adiponectin, as well as the macrophage-specific metabolite itaconate, which exert cancer-promoting effects by affecting inflammatory gene expression via various pathways [14,15]. This metabolic transition, mediated by obesity-related cytokines, or adipokines, functions as a stressor and promotes tissue damage that can promote neoplasia progression. Once a genetic mutation leads to oncogene activation, inflammation will contribute to cell proliferation, tumor establishment, growth, and metastasis. CRC is a cancer type known to be closely associated with chronic inflammation, making the progression to EOCRC a likely influence of these mediators [15,16].

EOCRC tumors have also been found to have distinct molecular features, compared to cancers found among older patient cohorts. In a study performed by Kothari et al., patients aged < 45 diagnosed with sporadic CRC were found to have upregulation in the FBXW7 gene, as well as mutations in the proofreading domain of polymerase ε catalytic subunit (POLE), with similar mutations in TP53, KRAS, and APC, compared to those > 65 years of age [17]. Further molecular analysis has also distinguished EOCRC from older patients, with an absence of BRAF mutations, methylator phenotypes, and implications of Wnt/beta catenin, MAP kinase, growth factor signaling, and tumor necrosis factor receptor 1 (TNFR1) pathways being highly prominent in younger cancer patients [18]. 

The aim of this review is to highlight current publications denoting the association of obesity-related hormones, inflammation, and the progression of EOCRC. The underlying mechanisms between specific adipokines and metabolites that have been studied and were thought to be crucial in the development of EOCRC are discussed. Further study is required to find associations between all components of the TME and EOCRC. 

## 2. Materials and Methods

A literature search was performed using PubMed, Cochrane, and Web of Science databases to identify studies related to obesity and EOCRC. Key words used included ‘EOCRC’, ‘obesity’, ‘obesity related hormones OR adipokines’, ‘itaconate’, ‘adiponectin’, ‘leptin’, ‘M2 macrophage’, and ‘microbiome’. A literature review was performed and studies that focused on describing the underlying inflammatory mechanisms of CRC development with relation to obesity-related hormones were included. The date of publication was set between 2013 and 2023. In this review, discussion centers on the above-mentioned regulators of obesity, adiponectin, leptin, and itaconate, and their role in the development of EOCRC.

## 3. Review

### 3.1. Obesity, Inflammation, and EOCRC

**Figure 2:**
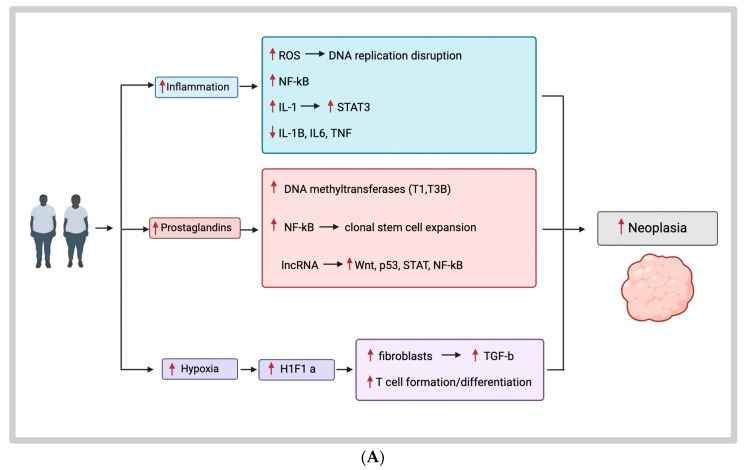

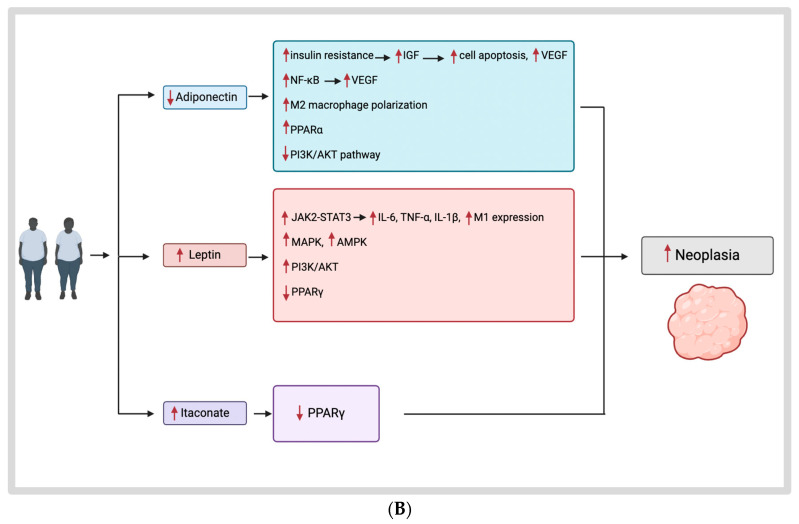
(**A**) Inflammation- and hypoxia-associated pathways in the progression of colorectal neoplasia. (**B**) Tumor-associated macrophage mediator pathways in the progression of colorectal neoplasia.

The mechanism behind inflammation and CRC is well understood, making inflammatory mediators a likely contributor to the development of CRC at earlier ages. Variants of CRC, including those developed via the sporadic, microsatellite instability pathway and other epigenetic mutations, have all been linked with inflammatory processes. These processes either precede tumor development, are the result of the tumors eliciting an inflammatory response in the host or are a result of therapeutic interventions [19]. In terms of inflammation-driven tumorigenesis, chronic inflammatory processes including inflammatory bowel diseases, infections, environmental factors such as smoking, and poor dietary habits among others have all been shown to increase the risk of CRC [19,20]. For example, in inflammatory colitis, the upregulation of monocyte chemoattractant protein-1 (MCP-1), an inflammatory chemokine, has been shown to be in higher concentrations in tumors versus normal mucosa and has also been the site of new targeted therapies to reduce cancer risk in inflammatory bowel disease [21,22]. Through various mouse models, the mechanism behind this development has been shown to require both an inciting event leading to epigenetic alterations, followed by tumor promotion, with inflammation contributing to both steps [19]. There are multiple mechanisms that lead to the formation of neoplasia in the setting of inflammatory states. Inflammation can cause disruption of DNA replication via the production of reactive oxygen and nitrogen species, leading to DNA strand breaks and nucleotide modifications (Figure 2A) [19,23]. Inflammation leading to DNA damage is a result of the activation of cytokine receptor-mediated signaling pathways, including activation of nuclear factor kappa-light-chain-enhancer of activated B cells (NF-κB), tumor necrosis factor (TNF), and interleukin-1 (IL-1) [19,24,25,26]. IL-1 is also a key inflammatory mediator expressed by stromal cells, monocytes, and tumor epithelial cells that not only drive the initiation but also the progression of cancer [27]. These processes contribute to the control of tumor initiation and promotion processes. IL-1 also activates STAT3 signaling. Lastly, inflammation can result in epigenetic changes that lead to the deactivation of important tumor suppressor genes such as IL-1β, IL-6, and TNF that control the regulation of DNA methyltransferases within the p53 and NOTCH pathways [19,28,29,30]. 

Prostaglandins are also upregulated during times of inflammation and have been shown to have a role in the development of all stages of CRC. Notably, Prostaglandin E2 (PGE2) promotes tumor initiation via activation of DNA methyltransferases T1 and T3B. PGE2 also increases clonal stem cell expansion via the NF-κB pathway and plays a role in the alteration of the TME to evade the immune response [31]. The prostaglandin pathway has become an area of interest in the prevention of CRC with the proposed use of COX inhibitors [32,33,34]. Inflammation has also been found to alter epigenetic pathways via the release of microRNAs and long non-coding RNAs [35]. These have been thought to target the WNT pathway, the Hippo pathway, as well as upregulating p53, and the STAT2 and NF-κB pathways [19,35,36,37,38,39,40]. 

Inflammation also damages the intestinal barrier leading to the exposure of intestinal stem cells to environmental pathogens, as well as increasing exposure to the gut microbiota, which contains bacteria with potential tumor-promoting characteristics. Another mechanism by which inflammation drives further tumor progression is via hypoxia and the recruitment of myeloid and lymphoid cells within the tumor microenvironment. Hypoxia induces the formation of hypoxia-inducible factor 1-alpha (HIF1α), leading to the activation of cancer-associated fibroblasts, with the release of transcription growth factor-beta (TGFβ) and other chemokines leading to the upregulation of cell diversity within the TME. Hypoxia caused by increased adipose tissue also reduces the natural immune defense by suppressing T-cell formation and differentiation [3,19]. Inflammation overall plays a key role in the development and progression of CRC. While the onset of CRC has been extensively linked to the development of inflammatory processes, not all these processes have yet been linked to the formation of EOCRC. However, the strong connection between the formation of sporadic colorectal cancer and inflammation is a reasonable hypothesis.

### 3.2. Obesity Hormones and Their Mechanisms

#### 3.2.1. Obesity and the Tumor Microenvironment

To unravel the complexity of mechanisms as potential therapeutic targets in EOCRC, it is crucial to understand the tumor microenvironment (TME). The TME consists of a network of cancer cells, stromal cells, extracellular matrix, and immune cells, specifically, tumor-associated macrophages (TAMs) [41]. TAMs make up one of the main tumor-infiltrating cell types within the TME [42]. They exist on a spectrum but have the ability to influence each other depending on TME changes. Two types of phenotypic macrophages have been described that are related to obesity and carcinogenesis: M1-like macrophages, which exert a mostly pro-inflammatory response, and M2-like macrophages, which act as mostly anti-inflammatory cells [41,43,44]. In obesity, there is an ongoing switch from the M2-like to the M1-like phenotype, causing downstream inflammatory responses. Furthermore, most studies show that the lack of the M2-like phenotype also correlates with obesity progression and inflammation [44]. There are many other cell types involved in the TME, including tumor-associated fibroblasts, endothelial cells, dendritic cells, lymphocytes, and other connective tissue cells; however, thus far, not all have been linked to the progression of EOCRC.

Obesity-related hormones are mostly secreted by adipocytes and have a direct impact on cytokine expression in CRC cells, as well as on M1/M2 macrophage polarization [14,41,45]. These TAMs, including both M1 and M2 macrophages, are key regulators of inflammatory signaling that have been found to affect CRC progression and patient survival. Mechanisms for TAM induction of neoplasms includes promoting tumor development by inducing interleukin 10 (IL-10) production in CRC cells through the STAT3 pathway, as well as upregulating production of cytokines such as vascular endothelial growth factor (VEGF) to induce tumor angiogenesis and tumor growth [46]. Advanced tumor stage and decreased overall/progression-free survival have been associated with TAMs, specifically, with a high M2/M1 ratio [41]. These macrophages also express several pro- and anti-inflammatory cytokines, such as interleukin-1β (IL-1β), interleukin-8 (IL-8), or interleukin-10 (IL-10), which increase the inflammatory state. The TME can also be affected by paracrine signaling and by the accumulation of systemic mediators. Pro-inflammatory M1 macrophages that produce reactive oxygen and nitrogen species may potentiate this effect, triggering oncogenic mutations in the adjacent epithelial layer [15,41]. Overall, TAMs, adipocytes, and cytokines all seem to play a central role in the development of EOCRC [46]. Leptin, adiponectin, and itaconate are mediators that have been found to either upregulate or downregulate the actions of TAMs via varying pathways.

Adipocytes secrete mediators known as adipokines which act on colonic cells to regulate metabolism [16]. The main two adipokines found to have tumor-promoting effects are leptin and adiponectin. Leptin and adiponectin are reported to have opposite roles in CRC progression, with leptin inducing cancer progression and adiponectin inhibiting tumor growth in vitro. Both leptin and adiponectin are secreted by white adipose tissue and affect macrophage polarization and cytokine expression [14,15,47,48]. As such, their regulation can alter cytokine profiles, resulting in either the promotion or inhibition of carcinogenesis. Not all mechanisms linking obesity, inflammation, and CRC apply to EOCRC. While there is a vast array of adipokines, such as omentin, resistin, apelin, vaspin, etc., not all their mechanisms of action have been recognized in reference to the development of EOCRC and they deserve continued study. The following are adipokines and chemokines that have been found to link the formation of obesity with earlier CRC onset.

#### 3.2.2. Adiponectin

Adiponectin is a 30 kDa complement C1q-related protein that plays a key role in insulin sensitivity and inflammation; it modulates metabolic functions such as glucose metabolism and fatty acid oxidation [48,49]. Decreased adiponectin levels have been associated with various forms of obesity-related malignancies, including gastric, uterine, breast, and CRC [48]. Experimental and population studies have found that low adiponectin levels, along with other hormonal alterations associated with metabolic syndrome, are involved in the development of carcinogenesis [49,50,51,52]. Reported mechanisms on CRC include the promotion of insulin resistance when adiponectin levels are low, leading to increased insulin growth factor-1 (IGF-1), which further promotes cellular proliferation, inhibits cell apoptosis, and upregulates VEGF (Figure 2). Adiponectin also has a more direct cancer-related role on TAMs that results in an increase in anti-inflammatory cytokine expression including IL-8. This increase in cytokine production occurs via an upregulation of the PPARα pathway and downregulation of NF-κB, which is a transcription factor that upregulates VEGF and IL-1β [49]. Adiponectin also mitigates the phosphatidylinositol-3-kinase/AKT (PI3K/AKT) pathway to limit carcinogenesis, tumor cell adhesion, and migration within CRC, with lower levels showing the opposite effects (Figure 2B) [47]. 

Within colon cancer, however, it has also been noted that adiponectin may have tumor-promoting effects via inducing more M2-like macrophage polarization which would favor CRC progression. In a study performed by Scheurlen et al., adiponectin was found to have tumor-promoting abilities by upregulating IL-8 expression and by inducing pro-inflammatory responses through tumor necrosis factor (TNF-α). When creating these M2a macrophages in vitro, they contradictorily become pro-inflammatory with production of inflammatory mediators, indicating in a certain setting they have a relatively different effect compared to their effects on regular monocyte and M1-like macrophages. Anti-inflammatory IL-8 production is a key factor in CRC development and is associated with enhanced tumor growth, progression, and recurrence [14]. Also, IL-8 promotes angiogenesis in CRC and regulates pro-inflammatory effects by functioning as a chemoattractant for neutrophils (Figure 2) [14,15]. Overall, this study demonstrated that the obesity-related hormone adiponectin may have a direct effect on gene expression patterns in CRC cells, leading to an enhancement of cancer progression rather than only downregulating it, as was previously reported [14]. 

#### 3.2.3. Leptin

The adipokine leptin circulates systemically as a 146-amino acid glycoprotein that is primarily produced and released by adipocytes [15,16]. Leptin, like adiponectin, has a metabolic impact, affecting cytokine expression in CRC, as well as macrophage polarization. It serves as a central mediator of inflammation, upregulating pro-inflammatory cytokine production such as interleukin 6 (IL-6), TNF-α, and IL-1β and increases M1-like marker expression in monocytes [14,47]. These cytokines serve as a link between obesity and inflammation with CRC carcinogenesis through TAM-mediated CRC cell progression. Leptin generates this upregulation by acting through the JAK2-STAT3 pathway, a key influence in tumorigenesis and metastasis (Figure 2) [15]. Via this pathway, leptin increases cell survival and cell growth in colon cancer cells [41,47]. The mitogen-activated protein (MAPK) and the AMP-activated protein kinase (AMPK) pathways are also regulated by leptin, both altering the gene transcription and inflammatory responses of colon cells [14,41]. The third mechanism by which leptin influences the progression of CRC is via PI3K/AKT signaling, where it has been found to induce immune suppression. This pathway is highly expressed in TAMs, but not in cancer cells. Within the tumor microenvironment, PI3K activation suppresses pro-inflammatory M1-like polarization of TAMs, creating an anti-inflammatory, tumor-promoting environment. Leptin activates AKT by phosphorylation, thereby affecting macrophage polarization and inducing TAM-mediated tumor progression [15]. The PPARγ pathway is yet another established mechanism in metabolic dysfunction and insulin resistance, and is also known as the glitazone receptor. Low PPARγ expression in CRC is associated with worse clinical outcomes [15,41,53,54]. Leptin slightly downregulates PPARγ expression in human macrophages, thereby contributing to this expression pattern [14,15].

All these pathways affect NF-κB activity, a key player in colorectal carcinogenesis. NF-κB is a transcription factor with multiple mechanisms, including the regulation of cytokine, cytokine receptor, and adhesion molecule expression in an inflammatory setting. NF-κB can affect both cancer cells and TAMs. The anti-apoptotic effect of leptin on cancer cells is also mediated through NF-κB inducing proliferation, differentiation, metastasis, angiogenesis, and chemoradiotherapy resistance in cancer cells. A high expression of leptin has overall been demonstrated to signify a poor prognosis in advanced-stage CRC [14]. 

### 3.3. Itaconate 

Itaconate is a macrophage metabolite that is produced by both M1-like macrophages and M2-like macrophages. M1-like macrophages supposedly produce this anti-inflammatory metabolite to self-regulate excessive inflammatory stress responses. However, M2-like macrophages can also produce itaconate under certain conditions [46]. Itaconate is produced during TAM polarization, and it is known to have tumor-promoting effects in ovarian carcinomas and gliomas [15]. Itaconate is produced by the expression of IRG1, a gene that encodes the protein aconitate decarboxylase 1 (ACOD1). ACOD1 is an enzyme of the tricarboxylic acid (TCA) cycle that produces the metabolite itaconate from cis-aconitate [45]. It serves as a regulator of cellular metabolism via the regulation of glycolysis and leads to succinate accumulation through the inhibition of succinate dehydrogenase. Itaconate also works to mediate anti-inflammatory effects by inducing anti-inflammatory transcription factors, such as nuclear factor erythroid-2 related factor 2 (NRF2), and oxidative stress reduction. It influences numerous transcription factors as well, including NF-κB, hypoxia-inducible factor 1α (HIF1α), signal transducer and activator of transcription 3 (STAT3), and activator protein 1 (AP-1) [14,15,41,45].

In vitro experiments have demonstrated the role of itaconate in the progression of EOCRC via its effects on inflammatory gene expression. IRG1 expression in macrophages is shown to be peroxisome PPARγ-dependent. Downregulation of PPARγ leads to increased IRG1 expression in peritoneal mouse macrophages, demonstrating that PPARγ is a regulator of macrophage metabolism (Figure 2). Additionally, PPARγ plays a pivotal role in epithelial cell differentiation, and decreased levels of PPARγ expression in CRC have been demonstrated to enhance CRC progression. Derivatives of itaconate that are lipid soluble, including 4-octyl itaconate (OI) and dimethyl itaconate (DI), exerted tumor-enhancing effects that were associated with poor patient survival by downregulating PPARγ, with OI having a slightly higher impact. Itaconate can reduce pro-inflammatory mechanisms and T-cell infiltration and, therefore, reduce anti-tumorigenic activity by downregulating CXCL10 [14]. This is a key factor of the anti-tumor T-cell response in EOCRC. 

### 3.4. Microbiome

Inflammatory processes and dysbiosis generated by the human microbiome are also a driving force behind the progression of colorectal neoplasia. This microbiome refers to the wide variety of microorganisms that reside in and on the human body. This flora consists of bacteria as well as fungi and viruses. Overall, a symbiotic relationship exists amongst this cohort and its human host, contributing to overall health but also contributing to human disease when dysregulated. There are more bacteria in the human body than human cells (3.8 × 10^13^ bacterial cells; 3.0 × 10^13^ human cells) [55]. The majority of these cells reside in the colon. Previous research has linked the dysbiota of the human microbiome to the development of intestinal neoplasia [56,57,58,59]. 

Bacteria commonly associated with the progression of both adenomas and CRC include Enteococcus coli, Bacteroides fragilis, and Lactobacillus casei. Alterations in the bacteria residing within the tumor microenvironment have also been associated with certain tumor characteristics such as tumor grade [60]. While each of these bacteria have multiple mechanisms within the tumor microenvironment, the majority of the bacteria linked to CRC involve the upregulation of inflammatory processes to generate neoplasia. Fusobacterium nucleatum is a prominent GI anaerobe that has been found to have a significant association with CRC. While it produces a biofilm, it also been shown to recruit tumor-infiltrating myeloid cells, as well as activate the NF-κB pathway leading to the production of inflammatory cytokines [61]. Enterococcus coli activates this same pathway, resulting in a similar effect [62]. Both bacteria also upregulate cellular proliferation and tumorigenesis via activation of the Wnt/βcatenin pathway [55]. Streptococcus gallactyus is an opportunistic bacterium which has been found to increase the release of pro-inflammatory markers such as IL-8, IL-1, and COX-2, which have been associated with the development of CRC [63,64]. Enterococcus faecalis is another common bacterium, associated with colitis, that serves to regulate the polarization of macrophages and influence the release of TNF-α [55,65]. Bacteriodes fragilis has been shown to induce colorectal tumorigenesis directly through its upregulation of inflammatory pathways. This occurs via the loss of the gut barrier function, which then generates the activation of the T-helper cell 17 inflammatory cascade, activation of the STAT3 pathway, activation of NF-κB signaling, and release of IL-17, a pro-inflammatory cytokine, with the upregulation of IL-17 receptors on colonic epithelial cells [66]. 

Dysregulation of the gut microbiome employs a diverse range of molecular mechanisms to promote the progression of CRC. The induction of inflammation is a contributor, either via direct or indirect methods, to promote the formation and progression of colorectal tumorigenesis. Both efforts to inhibit these inflammatory responses, as well as modulate bacteria within the gut microbiome, are currently under review as a means to reduce the formation of CRC [67,68,69,70,71]. 

In terms of EOCRC, a recent study performed by Barot et al. demonstrated that colorectal tumors arising in younger patients (median age 43) had distinct microbial profiles that were associated with tumor location, site of tumor, stage, and obesity level. Both the older adult cohort (median age 73) and younger cohort exhibited a core microbial profile consisting of Staphylococcus, Lactobacillus, Bacillus, Listeria, Akkermansia, Pseudomonas, Enterococcus, Alistipes, Escherichia/Shigella, and Fusobacterium compared to non-malignant tissue samples. Those within the EOCRC cohort, however, demonstrated a tumor microbiota with dysbiosis of both Bacteroides and Akkermansia compared with the older CRC patients [72]. Akkermansia is a mucin-degrading bacterium that promotes maintenance of the gut integrity barrier. It is known to have beneficial roles in autoimmune and chronic inflammatory diseases, with other findings showing that lower levels of Akkermansia are present in the gut of humans and mice with metabolic diseases [73,74,75,76]. When accounting for BMI as well, the tumor microbiome for those with a BMI > 25 also demonstrated a dysregulation of Limosilactobacillus, Listeria, Akkermansia, Enterococcus, and Escherichia/Shigella [72]. This increased level of diversity, as well as distinction within EOCRC microbiome pathogenesis, demonstrates a likely correlation between environmental/lifestyle factors and the upregulation of microbiota in response to inflammatory processes to protect the gut barrier integrity. The novelty of this dysbiosis could also signify the role of the microbiome in aiding the tumor microenvironment to evade the host defense systems [72]. In either scenario, continued understanding of the role of the microbiome in the development of EOCRC is an important area for future therapeutic interventions and possible preventions. 

## 4. Conclusions

The impact of obesity and metabolic dysfunction has been established in the development of EOCRC. Inflammation affects every aspect of tumor initiation and progression and leads to the modulation of various cells within the TME and the production of multiple cytokines. As a byproduct of TAM polarization, itaconate production has been found to upregulate the pathways of tumor progression. The adipokines leptin and adiponectin have also been found to affect various inflammatory markers, pathways within TAMs, and the tumor microenvironment that lead to carcinoma progression. Alterations to the gut microbiome leading to pro-inflammatory states have also been associated with the development of CRC, with its association with earlier-onset cancer providing a new opportunity for scientific inquiry. As EOCRC has been determined to be a distinct sub-type of CRC with unique molecular pathways regulating both initiation and progression, the continued evaluation of mediators affecting this sub-type remains crucial. As the tumor microenvironment associated with EOCRC is a very dynamic environment, it is hard to characterize the exact ways these mediators affect the progression of neoplasia. Continued experimentation and a better understanding of obesity-related hormone effects, the pathways they alter, and the association of pro-inflammatory cytokine expression within the TME of patients with EOCRC will provide additional opportunities for therapeutic targets to treat and prevent EOCRC.

## Data Availability

The data used in Figure 1 were obtained via Table 10 from the published work: [2].
